# Development of Multiexon Skipping Antisense Oligonucleotide Therapy for Duchenne Muscular Dystrophy

**DOI:** 10.1155/2013/402369

**Published:** 2013-07-31

**Authors:** Yoshitsugu Aoki, Toshifumi Yokota, Matthew J. A. Wood

**Affiliations:** ^1^Department of Physiology, Anatomy and Genetics, University of Oxford, South Parks Road, Oxford OX1 3QX, UK; ^2^Department of Medical Genetics, School of Human Development, Faculty of Medicine and Dentistry, University of Alberta, Edmonton, AB, Canada T6G 2H7; ^3^The Friends of Garrett Cumming Research and Muscular Dystrophy Canada HM Toupin Neurological Science Research Chair, University of Alberta, Edmonton, AB, Canada T6G 2H7

## Abstract

Duchenne muscular dystrophy (DMD) is an incurable, X-linked progressive muscle degenerative disorder that results from the absence of dystrophin protein and leads to premature death in affected individuals due to respiratory and/or cardiac failure typically by age of 30. Very recently the exciting prospect of an effective oligonucleotide therapy has emerged which restores dystrophin protein expression to affected tissues in DMD patients with highly promising data from a series of clinical trials. This therapeutic approach is highly mutation specific and thus is personalised. Therefore DMD has emerged as a model genetic disorder for understanding and overcoming of the challenges of developing personalised genetic medicines. One of the greatest weaknesses of the current oligonucleotide approach is that it is a mutation-specific therapy. To address this limitation, we have recently demonstrated that exons 45–55 skipping therapy has the potential to treat clusters of mutations that cause DMD, which could significantly reduce the number of compounds that would need to be developed in order to successfully treat all DMD patients. Here we discuss and review the latest preclinical work in this area as well as a variety of accompanying issues, including efficacy and potential toxicity of antisense oligonucleotides, prior to human clinical trials.

## 1. Introduction

Muscular dystrophy is a group of genetic diseases characterized by generalized progressive muscle atrophy and weakness and histopathology that reveals degeneration and regeneration of muscle fibres. In particular, Duchenne muscular dystrophy (DMD), the most common form of muscular dystrophy, is caused by lack of dystrophin protein [1]. In recent years, a most promising therapeutic approach to restoring dystrophin is antisense-oligonucleotide- (AO-) based exon skipping therapy that targets splicing or other elements in the dystrophin pre-mRNA [2]. This approach has been shown to be practical for up to 90% of DMD patients with deletion mutations, while read-through therapy with PTC124 is applicable for up to 10% of DMD patients with nonsense mutations [3, 4]. The principle underlying exon-skipping therapy for DMD has been demonstrated in cultured mouse, canine or human cells *in vitro *[5–11]. In addition, *in vivo* studies in murine or canine animal models have provided preclinical evidence for the therapeutic potential of AO-based exon-skipping strategies for DMD [12–23]. Finally, this therapeutic strategy has demonstrated efficacy in a number of recent phase I/II exon skipping trials which target exon 51 of the *DMD* gene [24, 25]. Currently phases II b and III exon skipping trials are ongoing by Sarepta Therapeutics and Prosensa/GlaxoSmithKline (GSK) with two different AO chemistries, phosphorodiamidate morpholino oligomers (PMO) and 2′O-methylated phosphorothioates (2′OMePs), respectively (ClinicalTrials.gov identifier: NCT01396239 and NCT01254019). It appears highly likely that AO-based therapeutic agents will receive regulatory approval for use in DMD within the next few years. Although promising, this therapeutic approach is highly mutation specific and thus is personalised. Therefore DMD has emerged as a model genetic disorder for understanding and overcoming the challenges associated with developing personalised genetic medicines [2]. This review highlights novel findings from a DMD mouse model utilizing systemic multiexon skipping targeting exons 45–55, discusses the major hurdles and limitations impeding the translation of this approach into clinical therapies along with the potential solutions, and provides a perspective on the future of clinical exon 45–55 skipping in DMD patients.

## 2. Duchenne Muscular Dystrophy

DMD, an incurable, X-linked progressive muscle degenerative disorder, presents with walking difficulties around 3–5 years of age [26]. Skeletal muscle involvement is chronically progressive, resulting in wheelchair-bound patients by the age of 13 years and being bed-bound thereafter. DMD patients usually die around the age of 30 years, typically from respiratory or cardiac failure due to dilated cardiomyopathy. DMD is most frequently caused by a frame-shift, nonsense, or duplication mutation in the *DMD* gene, which encodes dystrophin [27]. Dystrophin protein is localized at the subsarcolemma of muscle fibres and forms a dystrophin glycoprotein complex (DGC) with dystroglycans, sarcoglycans, syntrophins/dystrobrevins, sarcospan, and neuronal nitric oxide synthase (nNOS) complexes [28]. The DGC provides a mechanical and signaling link between the actin cytoskeleton and the extracellular matrix [29]. The absence of dystrophin leads to recurrent muscle fibre damage during contraction, and muscle fibres are eventually replaced by adipose and fibrotic tissues. Interestingly, a related allelic disorder, Becker muscular dystrophy (BMD), can exhibit a much milder phenotype. Many people with BMD live into their 70s and 80s [30]. BMD typically results from shortened, but in-frame, transcripts of the *DMD* gene that allow for expression of limited amounts of an internally truncated, but partially functional, protein (the reading frame rule) [31]. Interestingly, the severity of BMD can vary considerably from almost asymptomatic to a slightly less severe DMD-like condition [32–34].

## 3. Antisense Oligonucleotides Frequently Used in Exon Skipping

The development of AOs for manipulating alternative splicing has led to studies that focused on the therapeutic potential of AO-based exon skipping therapy for DMD. AOs can be designed to hybridize to a specific mRNA target and mediate its destruction via Ribonuclease H (RNase H), an enzyme that destroys mRNA in a DNA/RNA complex. However, the requirements for AOs that alter splicing are different from AOs used to achieve gene downregulation [35]. In this context, AOs can be used to modulate the ratio of splicing variants or to correct splicing defects, which has greater implications for treating a variety of diseases. In particular, the AOs should not activate RNase H, which would destroy the pre-mRNA before it can be spliced. In addition, the AOs must access the target pre-mRNAs within the cell nuclei to efficiently compete with splicing factors. Several types of modified synthetic AOs fit these criteria: AOs with backbones based on PMO [24, 36] and with modifications to the 2′ position of RNA, such as 2′OMePs [25, 37], are RNase H inactive and display high nuclease resistance and an affinity for target sequences. Similar characteristics are found in AOs with backbones based on phosphorothioate (PS) [38–40], peptide nucleic acid (PNA) [41, 42], locked nucleic acid (LNA) [43], and tricyclo-DNA [44]. However, a number of limiting factors have slowed the progress of AO drugs in the clinical area, including off target effects and low efficacy, partly due to delivery difficulty. The recent developments of second generation PMO have addressed many of the delivery issues and can, therefore, represent an effective strategy for reducing dose levels and frequencies, as well as delivery to nonleaky fibres [45, 46]. Vivo morpholino (vPMO), a nonpeptide transporter, comprises a dendritic structure assembled around a triazine core which serves to position eight guanidinium head groups in a conformation effective at penetrating cell membranes [47]. More recently, arginine rich, PMO-internalization-peptide- (Pip-) conjugated PMO has been developed [48]. Pips are characterized by a central hydrophobic motif flanked by arginine rich domains. Pip-PMO with improved cardiac exon skipping activity demonstrates highly efficient dystrophin protein expression in various muscles, resulting in up to 50% of wild-type levels of dystrophin protein in the heart in *mdx* mice [49].

## 4. Proof of Principle of AO-Based Exon Skipping for DMD Patients

The exciting prospect of an effective AO-based exon skipping to restore dystrophin protein expression to affected tissues in DMD patients has been backed up by highly promising data from a series of clinical trials. Firstly, efficient in-frame dystrophin expression and no clinically adverse events following an exon 51-skipping approach have been successfully demonstrated in human subjects using local intramuscular injection of 2′OMePs or PMO [50, 51]. Recently, systemic trials using both AOs have revealed dose-dependent efficacy without any severe adverse effects [24, 25]. Especially it is very promising that systemically injected 2′OMePs have shown benefits in walking distance [25, 52]. These study results are an important milestone, and Sarepta Therapeutics and Prosensa/GSK are now conducting a double-blind, placebo-controlled, II b and III exon skipping trials with PMO (ClinicalTrials.gov identifier: NCT01396239) and 2′OMePs (ClinicalTrials.gov identifier: NCT01254019).

## 5. Rationale of Exons 45–55 Skipping in DMD

**Figure 1:**
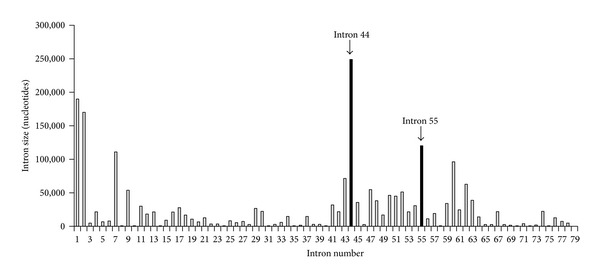
Intron sizes around exons 45–55 of the *DMD* gene. The intron size of exon 44 (intron 44) is 248,400 nucleotides; subsequent introns are shorter from 2,300 to 54,200 nucleotides, until intron 55, which is 120,200 nucleotides. Interestingly, we and another group have detected very minimal spontaneous exons 45–55 skipping at low frequency in mouse and human cells.

Although AO-based exon skipping is very promising, exon 51-skipping, which targets a single exon in the* DMD* gene and whose skipping can restore the open reading frame to the largest proportion of DMD mutations, is still applicable to only some 10% of DMD patients (examples of possible target mutations are as follows: deletions of exons 48–50, 45–50, 49-50, 50–52, 47–50, 43–50, and 52–63) [13, 53]. In addition, a phase I/IIa trial targeting exon 44 is currently underway (ClinicalTrials.gov identifier: NCT01037309) and development of new AO drugs targeting exons 45, 52, 53 and exon 55 is ongoing by Prosensa/GSK and Nippon Shinyaku Co., Ltd (UMIN000010964). By skipping these exons, approximately 28% of DMD patient mutations would be potentially treatable [54]. On the other hand, genotype and phenotype data from clinical databases suggest that simply restoring the reading frame may not necessarily result in restoring dystrophin protein function [55]. Two major hurdles of the current oligonucleotide approach are that (1) they are mutation specific and (2) the function or stability of each resulting in-frame dystrophin is uncertain. The prospect of overcoming both of these hurdles simultaneously has been inspired by the observation that patients with a deletion of exons 45–55 mostly show almost asymptomatic skeletal muscle involvement or exceptionally mild clinical symptoms with high blood creatine kinase concentrations, often associated with a late onset [56, 57]. It has also been noted that exons 45–55 cover the main mutation “hotspot” of the *DMD* gene so that, theoretically, up to 40–45% of DMD patients could be treated if it was possible to skip the entire exon 45–55 region [58]. Therefore, skipping of exons 45–55, that is, exons 45–55 skipping, at the mutation hotspot of the *DMD *gene with the second- generation peptide conjugated AOs would address these issues [27, 28]. Although multiexon skipping is technically very challenging, the existence of extremely mild patients with a spontaneous deletion of exons 45–55 implies that exons 45–55 skipping can be effective [12, 14]. Further the feasibility of exons 45–55 skipping can be explained by the order and timing of *DMD* intron removal. The *DMD* introns are extremely large compared to the mean size of human introns, 3,300 nucleotides [38, 39].  Figure 1 shows the intron sizes around exons 45–55 of the DMD gene. The intron 44 is 248,400 nucleotides. Subsequent introns are shorter (between 2,300 to 54,200 nucleotides), up until intron 55, which is 120,200 nucleotides. Interestingly, we and another group have detected very minimal spontaneous exons 45–55 skipping at low frequency in mouse and human cells, which may indicate that the acceptor splice sites of introns 44 and 55 can compete [17, 18, 59]. If the smaller introns (45 through 54) are indeed spliced out prior to intron 44 and intron 55, this would result in exons 45–55 skipping.

## 6. Dystrophin Molecular Structure and Function following Exons 45–55 Skipping

The molecular structure of dystrophin is composed of an actin-binding domain 1 at the N-terminus (ABD1), a central rod domain containing 24 spectrin-like repeats (R1–24), four hinge domains, a 20-amino acid insertion between spectrin-like repeats 15 and 16, a cysteine-rich domain, and a C-terminal domain [29]. Hinges are proline-rich, nonrepeat segments that possibly confer flexibility to dystrophin protein [60]. AO-based exon-skipping therapy for DMD is intended to exclude specific exons from out-of-frame dystrophin transcripts, thereby correcting the translational reading frame, resulting in the production of “BMD-like” in frame dystrophin, which would suppress nonsense-mediated mRNA decay and reconstruct the binding domain of dystrophin to DGC [11, 25]. The molecular structure of in-frame dystrophin as a result of exons 45–55 skipping leads to a truncation at the middle of two rod spectrin repeats (rod repeats 17 and 22) [16]. Interestingly, the number of rod repeats in this in-frame dystrophin between the remaining adjacent hinge domains H2 and H4 (16 spectrin repeats) is exactly the same as that between H2 and H3 in full-length dystrophin, which might indicate a requirement for such spacing for protein function or stability [55, 61]. It has been anticipated that the truncated dystrophin induced by exons 45–55 skipping would disturb the site-responsible anchoring of nNOS, which normally binds to spectrin-like repeats 16 and 17 of the dystrophin encoded by exons 42–45 and possibly render its subsarcolemmal localization unstable [62, 63]. It is encouraging to note that mild BMD patients with a deletion of exon 45–55 also lack nNOS at the subsarcolemma but have mild clinical symptoms (Table 1) [56, 58, 62, 64]. For example, it is reported that three patients with exon 45–55 deletions (28, 42, and 69 years old) showed no symptoms except for high blood CK level [56, 64]. That is, the loss of subsarcolemmal nNOS does not lead to a severe phenotype or that lack of nNOS does not appear to be critical. However, animal models should nonetheless be used to investigate the molecular function of in-frame dystrophin following exons 45–55 skipping [62].

## 7. Preclinical Exons 45–55 Skipping in the Exon 52 Deficient Muscular Dystrophy Mouse

**Figure 2:**
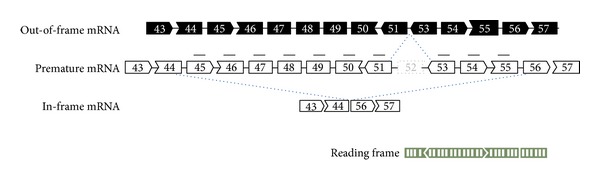
Exons 45–55 skipping with a mixture of 10 vPMOs in *mdx*52 mouse. *Mdx*52 mouse lacks exon 52 in the mRNA of the murine *DMD* gene, leading to out-of-frame products. Exons 45–55 skipping with mixture vPMOs (black line) restores the reading frame of *DMD* mRNA.

To date, a proof of concept for systemic exons 6 and 8 skipping by targeting two exons has been provided in dystrophic dogs [17, 18, 65]. However, exons 45–55 skipping with a mixture of 2′OMePs in DMD myoblast cells has proved technically challenging, and the levels of exons 45–55 skipping have typically been low and highly variable [59, 66]. Recently Takeda's group tested the feasibility of exons 45–55 skipping using both mouse myoblast cells and systemic injections with a mixture of 10 vPMOs in *mdx*52 mice, which harbour a deletion mutation in exon 52 [16, 67, 68] (Figure 2). The mixture of vPMOs was newly designed, paying specific attention to the avoidance of formation of self- or heteroduplex of the AOs, which might diminish the efficacy of exons 45–55 skipping. With this aim, we used OligoAnalyzer 3.1 to design the mixture in which most of combinations of Δ*G* maximum binding forces of two AOs were above −5 kcal/mole [69]. Efficient skipping of all of 10 exons was demonstrated with mixtures of 10 vPMOs* in vitro* in conditionally immortalized muscle cells called H2K-*mdx*52 myotubes. Moreover, on systemic injections into *mdx*52 mice with the mixtures (a total of 12 mg/kg dose), extensive dystrophin-positive fibres and an average of some 8–15% of wild-type levels of exons 45–55 truncated dystrophin protein as determined by Western blotting analysis in a range of skeletal muscles were observed, whereas the dystrophin expression level in the heart was only 2% of wild-type levels. The in-frame dystrophin restored all of the DGC except nNOS at the subsarcolemma. The pathology of skeletal muscles was ameliorated, but the skeletal muscle function was only marginally recovered, probably due to the incomplete restitution of the dystrophin. Although the sequences are specific to the mouse, these data validate the principle that carefully designed AOs may be used to realize exons 45–55 skipping and, by this means, generate effective amounts of in-frame dystrophin of near-optimal structure in 40–45% of DMD patients.

## 8. Towards Clinical Application: Hurdles and Limitations

Although results from preclinical trials appear encouraging, several issues pose challenges for the use of an AO “mixture approach” as an effective and affordable therapy for DMD. The most significant issue is in developing new approaches to toxicity testing and clinical trial regulatory procedures that are relevant and appropriate for sequence-specific mixture drugs [70]. It is important to note that mixtures of AOs might be considered one drug by drug regulatory agencies including those of the EU, USA, and Japan, even if the mixture contains several different AOs. Otherwise, required pre-clinical toxicology studies to develop this “mixture approach” will be too complicated and the costs for development of each of the 10 AOs and the subsequent clinical trials will be enormous. The second significant issue to translate the “mixture approach” with vPMOs to a therapy for DMD is the lack of adequate delivery to skeletal muscle and especially to the heart muscle. Higher dose administration of vPMOs would likely cause acute toxicity due to their narrow therapeutic range. Transient reduced activity, bradypnea, or seizure was sometimes observed following systemic injection of the mixture with vPMOs at a total of 15–25 mg/kg/dose in *mdx*52 mice. Importantly, cardiomyopathy is the second leading cause of death in DMD patients, accounting for 10–40% of deaths in DMD populations [71–73]. While some BMD patients with an exon 45–55 deletion show X-linked dilated cardiomyopathy [56, 58, 64, 74], their prognoses are quite favorable and only a few of them show mild heart failure symptom [62]. These imply that cardiac dystrophin correction due to exons 45–55 skipping would effectively rescue cardiac symptoms. The causes of low efficiency of cardiac dystrophin restoration remain unclear but are likely related to the inadequate ability of vPMO to penetrate the heart.To further validate and develop this exons 45–55 skipping approach, there are two critical steps, first to understand the order of *DMD* intron removal and the minimum combinatorial requirements for multiexon skipping of exons 45–55 (i.e., what is the fewest number of AOs that can be used to generate effective skipping). Secondly, this approach must now be combined with advanced generation peptide conjugated AO chemistry to achieve the maximum therapeutic outcome without toxicity for skeletal and cardiac muscles.

## 9. Conclusion

Recently, the prospect of successful AO-based therapies has moved a step closer to clinical applicability, in particular for DMD. The first clinical trials in DMD patients demonstrated the proof of principle of exon 51 skipping in humans, resulting in very encouraging results. However, multiple exon skipping, including exons 45–55 skipping using a “mixture approach”, is still in the preclinical stage. A variety of issues accompany this approach, including low efficacy and potential high toxicity of AOs, prior to human clinical trials. Development of an advanced exons 45–55 skipping approach with fewer new generation AOs that have improved cardiac exon skipping activity may reduce the therapeutic dose and interval of administrations, minimising the potential toxicity, off-target effects, and the cost burden for DMD.

## Figures and Tables

**Table 1:** Summary of clinical features of patients with an exon 45–55 deletion mutation of the *DMD* gene.

Patient ID	Age at onset of symptoms	Symptoms at onset	Clinical status	Dilated cardiomyopathy	Ambulant	Reference
1	2	Weakness	Mild BMD	not available	+	[58]
2	3.5	Weakness	Exercise intolerance	−	+	[58]
3	3.5	By chance	Asymptomatic	+	+	[58]
4	4	Weakness	Mild BMD	not available	+	[58]
5	4	Weakness	Mild BMD	not available	+	[58]
6	4	Fatigue, cramps after exercise	Mild BMD	−	+	[62]
7	4	Fatigue, mild difficulty in running	Mild BMD	−	+	[62]
8	6	Hyper-Ck-emia	Asymptomatic	−	+	[58]
9	8	Hyper-Ck-emia	Asymptomatic	−	+	[58]
10	9	Myoglobinuria	Mild BMD	−	+	[62]
11	12	Myalgia	Myalgia	not available	+	[58]
12	12	Exercise intolerance	Mild BMD	−	+	[58]
13	13	Muscle pain	Mild BMD	−	+	[58]
14	13	Hyper-Ck-emia	Mild BMD	−	+	[58]
15	19	Weakness	Mild BMD	−	+	[58]
16	26	Exertional dyspnea	No weakness and atrophy	+	+	[64]
17	36	Exertional dyspnea	No weakness and atrophy	+	+	[64]
18	40	Calf hypertrophy	Mild BMD	+	+	[58]
19	45	Weakness	Mild BMD	not available	+	[58]
20	49	Weakness	Mild BMD	not available	+	[58]
21	55	Walking difficulties	Mild BMD	−	+	[62]
22	59	Weakness	Mild BMD	−	+	[64]
23	69	Hyper-Ck-emia	Asymptomatic	−	+	[56]
